# Proton irradiation orchestrates macrophage reprogramming through NFκB signaling

**DOI:** 10.1038/s41419-018-0757-9

**Published:** 2018-06-27

**Authors:** Géraldine Genard, Anne-Catherine Wera, Camille Huart, Benjamin Le Calve, Sébastien Penninckx, Antoine Fattaccioli, Tijani Tabarrant, Catherine Demazy, Noëlle Ninane, Anne-Catherine Heuskin, Stéphane Lucas, Carine Michiels

**Affiliations:** 1 Cellular Biology Research Unit (URBC)—NARILIS, University of Namur, Namur, Belgium; 2 Laboratory of Analysis by Nuclear Reaction (LARN / PMR)—NARILIS, University of Namur, Namur, Belgium

## Abstract

Tumor-associated macrophages (TAMs) represent potential targets for anticancer treatments as these cells play critical roles in tumor progression and frequently antagonize the response to treatments. TAMs are usually associated to an M2-like phenotype, characterized by anti-inflammatory and protumoral properties. This phenotype contrasts with the M1-like macrophages, which exhibits proinflammatory, phagocytic, and antitumoral functions. As macrophages hold a high plasticity, strategies to orchestrate the reprogramming of M2-like TAMs towards a M1 antitumor phenotype offer potential therapeutic benefits. One of the most used anticancer treatments is the conventional X-ray radiotherapy (RT), but this therapy failed to reprogram TAMs towards an M1 phenotype. While protontherapy is more and more used in clinic to circumvent the side effects of conventional RT, the effects of proton irradiation on macrophages have not been investigated yet. Here we showed that M1 macrophages (THP-1 cell line) were more resistant to proton irradiation than unpolarized (M0) and M2 macrophages, which correlated with differential DNA damage detection. Moreover, proton irradiation-induced macrophage reprogramming from M2 to a mixed M1/M2 phenotype. This reprogramming required the nuclear translocation of NFκB p65 subunit as the inhibition of IκBα phosphorylation completely reverted the macrophage re-education. Altogether, the results suggest that proton irradiation promotes NFκB-mediated macrophage polarization towards M1 and opens new perspectives for macrophage targeting with charged particle therapy.

## Introduction

The immune system takes part in both cancer elimination and tumor development, especially through its activation and then its adaption to cancer cells^1^. This process is called the cancer immunoediting and it perfectly illustrates the ambivalent function of the immune system in cancer. In addition, the effectiveness of treatments such as X-ray or γ-ray radiotherapy (RT) is partially conditioned by the presence of immune cells into the tumor. For example, ablative radiation (20 Gy) on local tumor significantly reduced the tumor volume in wild-type (WT) but not in nude (T-cell-deficient) mice bearing a melanoma (B16 melanoma cells)^2^. In line with this observation, local RT produces an abscopal response, which is an antitumor effect on distant unirradiated tumors (metastases), by triggering systemic immune responses^3^. Another example is the systemic immune activation in mice previously treated by local RT and immune checkpoint inhibitors that prevented the tumor growth in mice rechallenged with the same tumor^4^. However, the number of infiltrating tumor-associated macrophages (TAMs) is known to limit radiotherapy efficiency and is directly correlated to a poor prognosis^5^. Inside the tumor, TAMs represent up to 50% of the host infiltrating cells^6, 7^, meaning that these cells are highly recruited into the tumor site and play critical roles in tumor development. It has been shown that RT promotes the recruitment of macrophages into the tumor, favoring tumor relapse after treatment^8^. Indeed, TAM depletion or inhibition of monocyte recruitment into the tumor site combined to RT induce tumor regression in mouse cancer models and increase the survival of cancer patients^9, 10^. Another attractive and effective strategy is the re-education of TAMs towards an antitumoral phenotype.

Macrophages display a remarkable plasticity allowing them to fulfill a multiple range of functions. These multitask skills rely on two opposite phenotypes, M1 versus M2. These two subpopulations are classified as two extremes of a linear scale between which exists a multitude of intermediate states^11^. Proinflammatory M1 macrophages, also called classically activated macrophages, exhibit enhanced pathogen phagocytosis, promote inflammation, and activate immune system. On the opposite, anti-inflammatory M2 macrophages, referred to as alternatively activated macrophages, contribute to tissue repair, matrix remodeling, and angiogenesis, and also repress the immune system. It is well accepted that M1 macrophages exert antitumor functions while M2 macrophages show protumoral activity and, unfortunately, are usually the most representative TAM population into the tumor^12^. M2-like TAMs display their harmful actions by promoting genetic instability, stem cell nurturing, angiogenesis, metastasis spreading, and local immunosuppression^8^. The polarization of macrophages towards an M1 or an M2 phenotype is driven by the activity of diverse transcription factors and miRNAs. Among transcription factors, NFκB plays a central role to influence the inflammatory macrophage status. While the active heterodimer NFκB (p50–p65) promotes the transcription of proinflammatory genes, such as TNFα, IL-6, and IL1β, the inactive homodimer NFκB (p50–p50) prevents the transcription of proinflammatory genes and confers the anti-inflammatory status to M2 macrophages^13^.

The plasticity of TAMs and their ability to be reprogrammed, especially from M2 to M1 phenotype, make them an attractive target for anticancer therapies. Conventional radiotherapy (X-rays or γ-rays) initiated the polarization of differentiated but unpolarized (M0) macrophages towards M1 when exposed to moderate doses (1–10 Gy) (for a review, see ref. ^14^). In addition, the combination of CD8^+^ T-cell transfer and γ-ray used at moderate doses (2 Gy) re-educated TAMs towards an M1 phenotype in a pancreatic tumor mice model^10^. Other studies also reported TAM reprogramming after low dose of whole-body irradiation^15, 16^. However, no study has established the reprogramming of M2 into M1 macrophages with local conventional RT only. This suggests that RT alone is not sufficient to reverse macrophage polarization.

Over the last decades, efforts were aimed to improve the delivery of conventional RT using image guidance. Despite these improvements, side effects associated to this treatment have remained severe. To spare surrounding healthy tissues, protontherapy presents an increasing interest, thanks to the charged nature of the particles and its depth dose profile. In more details, the one-shot energy release at the end of the charged particle track allows the improvement of dose conformation. This is added to the fact that the track of charged particles can be easily deviated by a magnetic field to precisely target the tumor. As for X-ray irradiation, the deposited energy by charged particle beam promotes the ionization of DNA through reactive oxide species (ROS) production. In addition to these indirect DNA damage, charged particles also directly interact with DNA, resulting in more complex DNA damage^17^. Our work demonstrated the ability of protontherapy to induce macrophage reprogramming. By using THP-1-derived M0, M1 and M2 macrophages, we evidenced that proton irradiation, but not X-ray irradiation, induced a partial switch from M2 to M1 macrophages. This macrophage reprogramming is orchestrated, at least in part, by an NFκB activation.

## Results

**Fig. 1:**
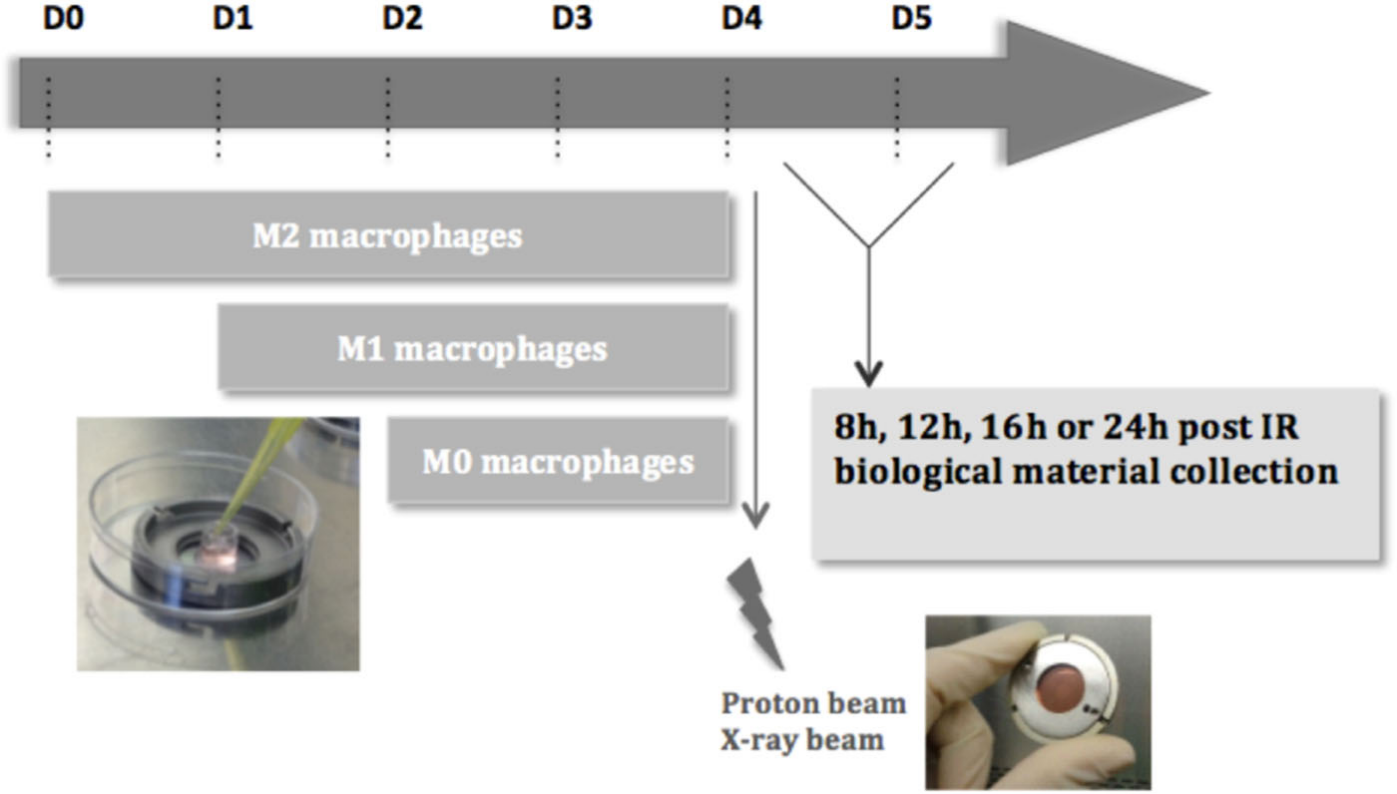
Schematic outline of the irradiation procedure for macrophages. THP-1 monocytes were differentiated into macrophages (M0) with 150 nM PMA in cloning cylinder, placed at the center of the irradiation chamber. Macrophages were polarized in M1 phenotype with 10 pg/ml LPS and 20 ng/ml IFN-γ during 24 h incubation or were polarized in M2 phenotype with 20 ng/ml IL-4 and IL-13 during 48 h incubation. THP-1 monocytes were differentiated on the appropriate day, in order to obtain the three phenotypes on day 4. Following the experiment that was performed, the biological material was collected 8, 12, 16, or 24 h after proton or X-ray irradiation

To address these goals, THP-1 cells were differentiated (M0) and polarized (M1 or M2) in irradiation chambers (Fig. 1). These special devices were placed at the end of the accelerator-produced proton beam and the effects on macrophages were then analyzed.

### M1 macrophages are more resistant to moderate doses of proton irradiation than M0 and M2 macrophages

**Fig. 2:**
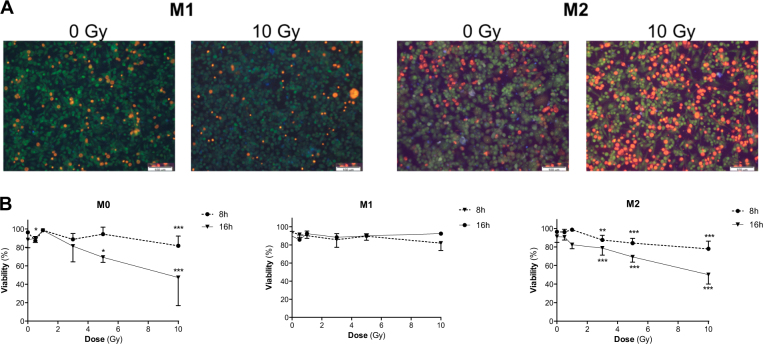
Early radioresistance of M1 macrophages after moderate doses of proton irradiation. M0, M1, and M2 macrophages were irradiated with different doses of protons. Viability was assessed by ethidium bromide−acridin orange at different times postirradiation. **a** Representative ethidium bromide−acridine orange staining images of M1 and M2 macrophages 16 h after proton irradiation (0 Gy and 10 Gy). Lived cells appeared in green while dead cells are stained in orange. **b** Quantification of viability (%) in M0, M1, and M2 macrophages, 8 or 16 h after proton irradiation. *N* = 3 for each dose (mean ± SD). One-way ANOVA analyses followed by Dunnett’s multiple comparisons post-tests were performed on data; *p ≤ 0.05; **p < 0.01; ***p < 0.001

In order to evaluate the cell viability after proton irradiation, ethidium bromide−acridine orange staining was performed (Fig. 2a) and the number of dead cells (orange) and viable cells (green) was counted (Fig. 2b). The cell viability slightly decreased 8 h (Fig. 2b) after moderate proton irradiation doses (0–10 Gy), as indicated by a survival of 82% for M0 macrophages, 82% for M1 macrophages, and 78% for M2 macrophages when irradiated with a dose of 10 Gy. Proton irradiation at 10 Gy further lowered the viability of M0 and M2 macrophages respectively to 42 and 50% after 16 h (Fig. 2b). Surprisingly, the viability of M1 macrophages was only slightly affected (92%) 16 h after proton irradiation at doses as high as 10 Gy. These results suggest an early radioresistance of the M1 phenotype to moderate proton irradiation doses, compared to the two other phenotypes.

### M1 radioresistance correlates with more intense γH_2_AX and 53BP1 labeling

**Fig. 3:**
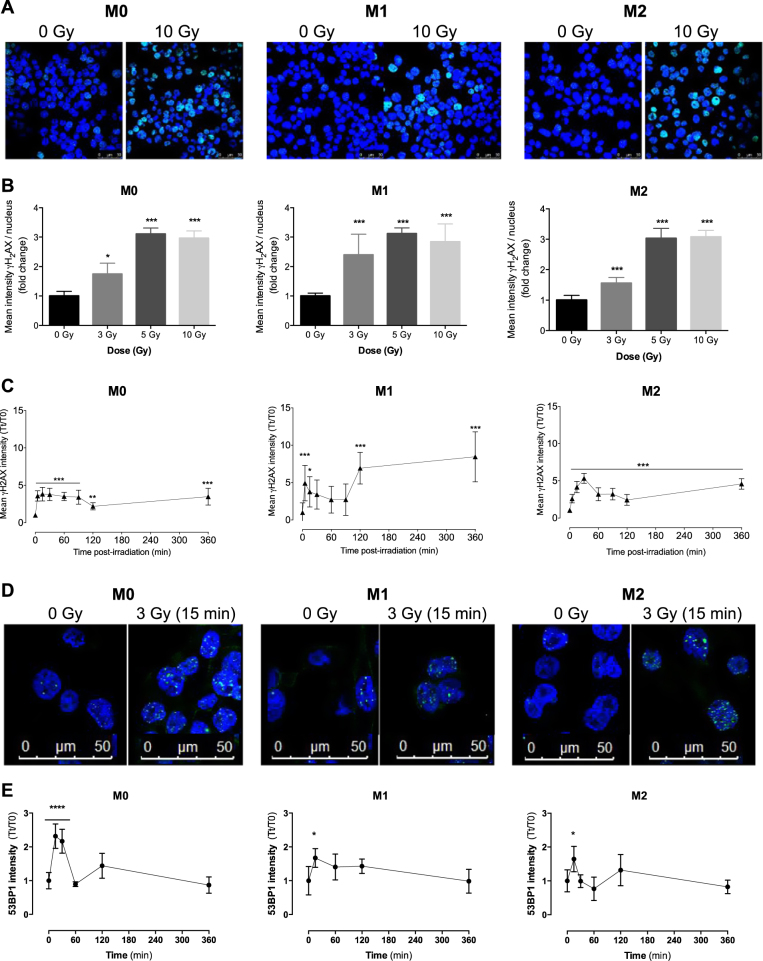
M1 resistance to proton irradiation correlates with a higher γH_2_AX and 53BP1 labeling. M0, M1, and M2 macrophages were irradiated with different doses of protons. The evaluation of DNA damage following proton irradiation was performed by phosphorylated H_2_AX (γH_2_AX) or 53BP1 labeling. **a** Representative immunofluorescence labeling of γH_2_AX for M0, M1, and M2 macrophages 15 min after proton irradiation. γH_2_AX labeling appears in green and nuclei are stained in blue (To-pro). **b** Quantification of the mean γH_2_AX intensity per nucleus 15 min after proton irradiation. Results are expressed in mean pixel intensity value and are normalized to the nonirradiated condition (fold change). Quantifications were performed on minimum five images per condition; representative experiment (*N* = 3, mean ± SD). **c** Mean γH_2_AX intensity after proton irradiation (3 Gy), several times postirradiation. Each point corresponds to the mean γH_2_AX intensity at time *t* (*Tt*) normalized to time 0 (*T*0). Quantifications were performed on minimum ten images per condition; representative experiment, *N* = 3 (mean ± SD). One-way ANOVA analyses were performed on data, followed by Dunnett’s post-tests; *p ≤ 0.05; **p < 0.01; ***p < 0.001. **d** Representative immunofluorescence labeling of 53BP1 for M0, M1, and M2 macrophages 15 min after proton irradiation. 53BP1 labeling appears in green and nuclei are stained in blue (To-pro). **e** Quantification of the 53BP1 intensity per nucleus after proton irradiation (3 Gy), several times postirradiation. Each point corresponds to the mean 53BP1 intensity at time *t* (*Tt*), normalized to time 0 (*T*0). Quantifications were performed on five images per condition; *N* = 1 (mean ± SD). One-way ANOVA analyses were performed on data, followed by Dunnett’s post-tests; *p ≤ 0.05; **p < 0.01; ***p < 0.001

In order to further examine the influence of proton irradiation on macrophages, phosphorylated H_2_AX (Ser 139) (γH_2_AX), a sensitive marker for DNA double-strand breaks (DSBs), was evaluated by immunofluorescence labeling (Fig. 3a). Quantifications of γH_2_AX labeling (Fig. 3b) 15 min after irradiation indicated a similar profile for the three macrophage phenotypes when irradiated at different doses (3, 5, and 10 Gy). However, the quantifications of γH_2_AX labeling (Fig. 3c) over the time post irradiation (0–360 min) indicated a similar profile for M0 and M2 macrophages while M1 phenotype exhibited a higher level of phosphorylated H_2_AX. In more details, γH_2_AX labeling increased 5 min after proton irradiation (3 Gy) in M0 and M2 phenotypes and was mostly decreased 2 h after irradiation. In contrast, the γH_2_AX labeling was increased and plateaued as long as 6 h after irradiation in M1 macrophages, indicating an increasing and prolonged detection of DSBs in this phenotype. In order to confirm these data, 53BP1 intensity was also assessed by immunofluorescence labeling (Fig. 3d). 53BP1 is known for its role in DNA repair machinery. Similarly to γH_2_AX labeling, the quantification of 53BP1 intensity revealed a sustained labeling over the time in M1 macrophages, while 53BP1 intensity was decreased 1 h after proton irradiation in M0 and M2 macrophages (Fig. 3e). As chromatin conformation could influence the detection and the repair of DSBs, we evaluated the heterochromatin content by MNAse I assay in the three different phenotypes. The radioresistance of M1 macrophages was not linked to a higher level of DNA condensation before irradiation since there was no difference in the heterochromatin content in the three phenotypes (Fig. S1). In addition to sustained γH_2_AX, the analysis of ROS content by flow cytometry revealed that M1 macrophages better managed H_2_O_2_ treatment (Fig. S2). Taken together, these results indicated that the radioresistance of M1 macrophages to proton irradiation could be related to higher DSB detection and/or to better efficiency of DNA repair machinery in this phenotype, and also correlates with a better elimination of ROS.

### Proton irradiation induces macrophage reprogramming

**Fig. 4:**
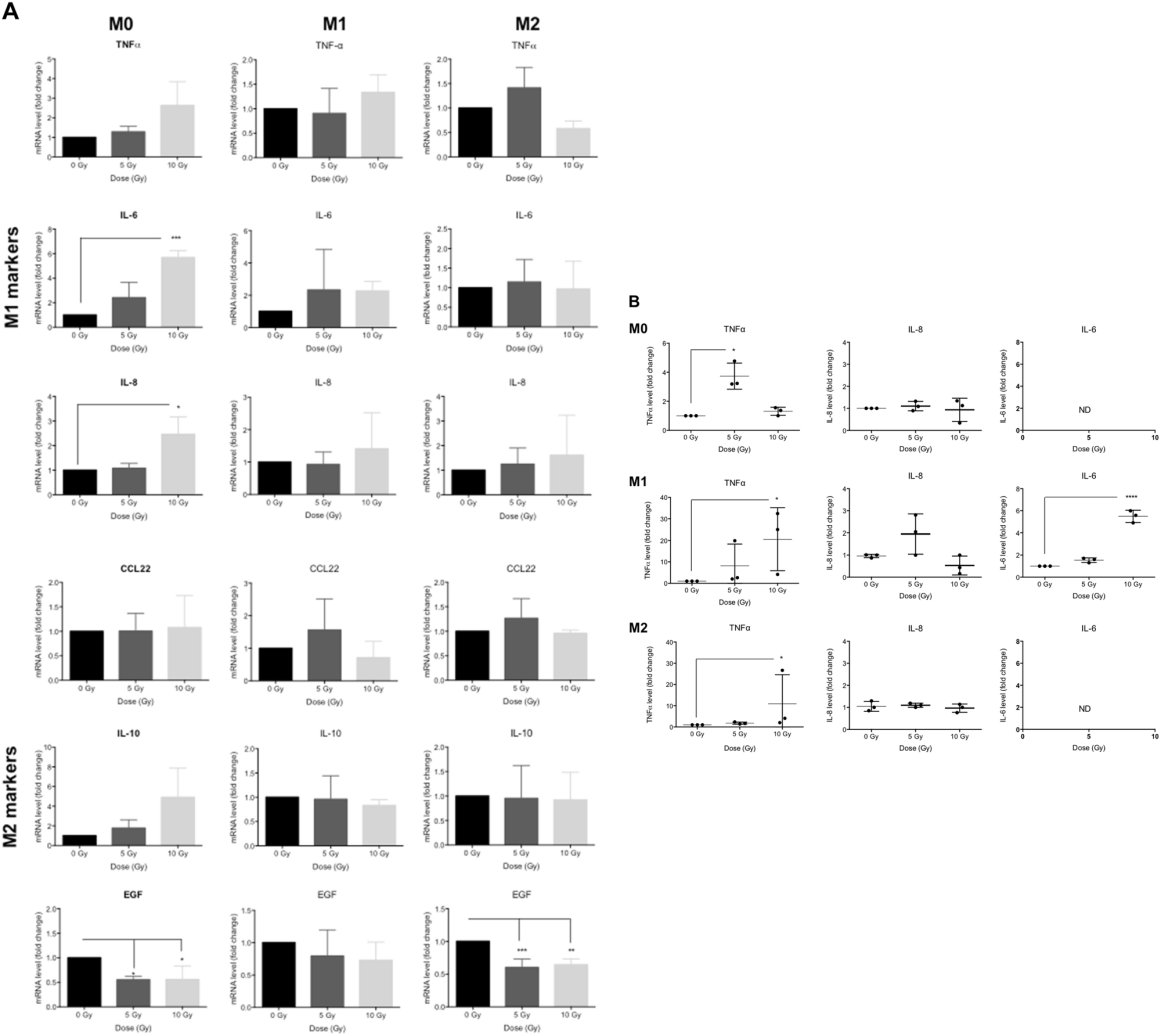
Proton irradiation induces macrophage reprogramming. M0, M1, and M2 macrophages were irradiated with different doses of protons (0, 5, and 10 Gy). **a** 24 h after proton irradiation, mRNA levels of M1 (TNFα, IL-6, IL-8) and M2 (CCL22, IL-10, EGF) markers were assessed by RT-qPCR (*N* = 3, mean ± SD). One-way ANOVA analyses followed by Dunnett’s multiple comparison tests were performed to evaluate the significance (*p ≤ 0.05; **p < 0.01; ***p < 0.001). **b** 24 h after proton irradiation, TNFα, IL-8, and IL-6 secretion was evaluated by ELISA. Results are expressed in pg/ng of proteins and are normalized to the nonirradiated condition (fold change) for each macrophage phenotype; ND was used for not detected (*N* = 3, mean ± SD). One-way ANOVA analyses followed by Kruskal−Wallis multiple comparison tests were performed on data (*p ≤ 0.05; **p < 0.01; ***p < 0.001)

Proton irradiation of M0, M1, and M2 macrophages with moderate doses promotes the reprogramming of M0 and M2 macrophages towards an M1 phenotype (Fig. 4). mRNA levels of several M1 markers (IL-6 and IL-8) increased significantly in M0 macrophages (Fig. 4a, left panel) exposed to 10 Gy. At the same time, we observed a significant reduction of the mRNA expression of EGF, a specific marker of M2 phenotype. Consistent with these results, the secretion of TNFα was also significantly higher (Fig. 4b, left panel) in this macrophage phenotype after 5 Gy of proton irradiation. Taken together, these results indicated a polarization of M0 macrophages towards a M1 phenotype after moderate doses of proton irradiation. The irradiation of M1 macrophages (Fig. 4a, middle panel) did not exhibit any change in mRNA expression for M1 (TNFα, IL-6 and IL-8) and M2 (CCL22, IL-10 and EGF) markers. The secretion of IL-6 and TNFα was then quantified after moderate doses of irradiation (Fig. 4b, middle panel). The results revealed a strengthening of the M1 phenotype in M1 macrophages. The proton irradiation of M2 macrophages (Fig. 4a, right panel) led to a significant decrease in EGF mRNA expression at 5 and 10 Gy while the expression of other M2 markers remained unchanged. The mRNA level of M1 markers was not affected by proton irradiation. However, the irradiation of M2 macrophages generated a significant increase in TNFα secretion (Fig. 4b, right panel). As a whole, proton irradiation (5 and 10 Gy) initiated the polarization of M2 macrophages towards the M1 phenotype, thus generating an intermediate phenotype. Similarly to other studies, X-ray irradiation did not succeed to induce a reprogramming of M2 macrophages towards an M1 phenotype in our model (Fig. S3). The mRNA expression of M1 and M2 markers was not affected by X-ray irradiation in M0 and M2 macrophages, while the mRNA level of EGF was reduced in irradiated M1 macrophages. In addition, the secretion of TNFα was elevated in irradiated M1 macrophages, consistent with a strengthening of the M1 phenotype in M1 macrophages.

### Proton irradiation orchestrates NFκB p65 nuclear translocation in macrophages

**Fig. 5:**
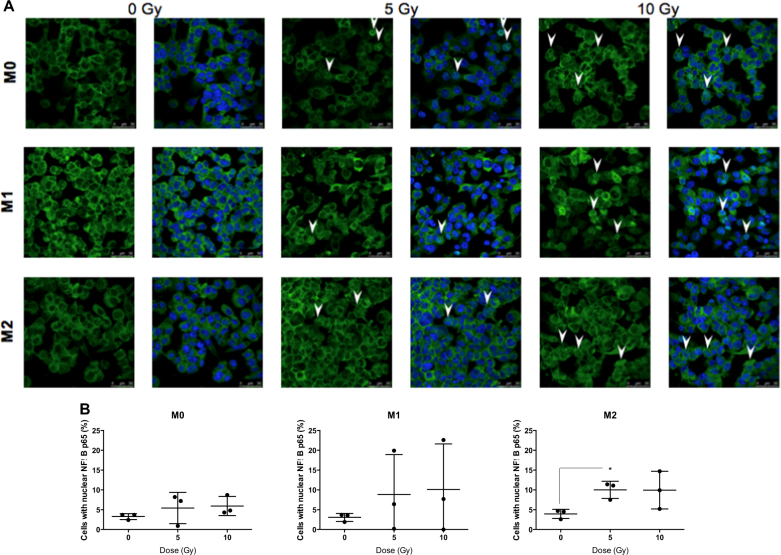
Proton irradiation induces nuclear translocation of p65 (NFκB). M0, M1, and M2 macrophages were irradiated by different doses of protons. 2 h after the irradiation, the nuclear translocation of p65 was evaluated by NFκB p65 immunofluorescence labeling. NFκB p65 is stained in green and nucleus appears in blue. **a** Nuclear translocation of NFκB p65 is indicated (arrow) on representative immunofluorescence labeling images for M0, M1, and M2 macrophages after irradiation. **b** Quantification of the mean NFκB p65 intensity per nucleus 2 h after proton irradiation. Results are expressed in percentage of cells with nuclear NFκB p65. Quantifications were performed on minimum five images per condition (*N* = 3, mean ± SD). An unpaired *t* test was performed on data (*p ≤ 0.05; **p < 0.01; ***p < 0.001)

To investigate the mechanism underlying macrophage reprogramming after moderate doses of proton irradiation, the nuclear translocation of NFκB p65 subunit was evaluated after the irradiation. This translocation was analyzed in M0, M1, and M2 macrophages at different times postirradiation (0, 5, 15, 30, 60, 90, 120, and 360 min) (data not shown) and was observed in the three phenotypes 2 h after the irradiation (Fig. 5a). The quantification of highly positive cells for NFκB p65 suggested a nuclear translocation of NFκB p65 in M0, M1, and M2 macrophages after proton irradiation (5 and 10 Gy) (Fig. 5b).

### NFκB inhibition reverts macrophage reprogramming induced by proton irradiation

**Fig. 6:**
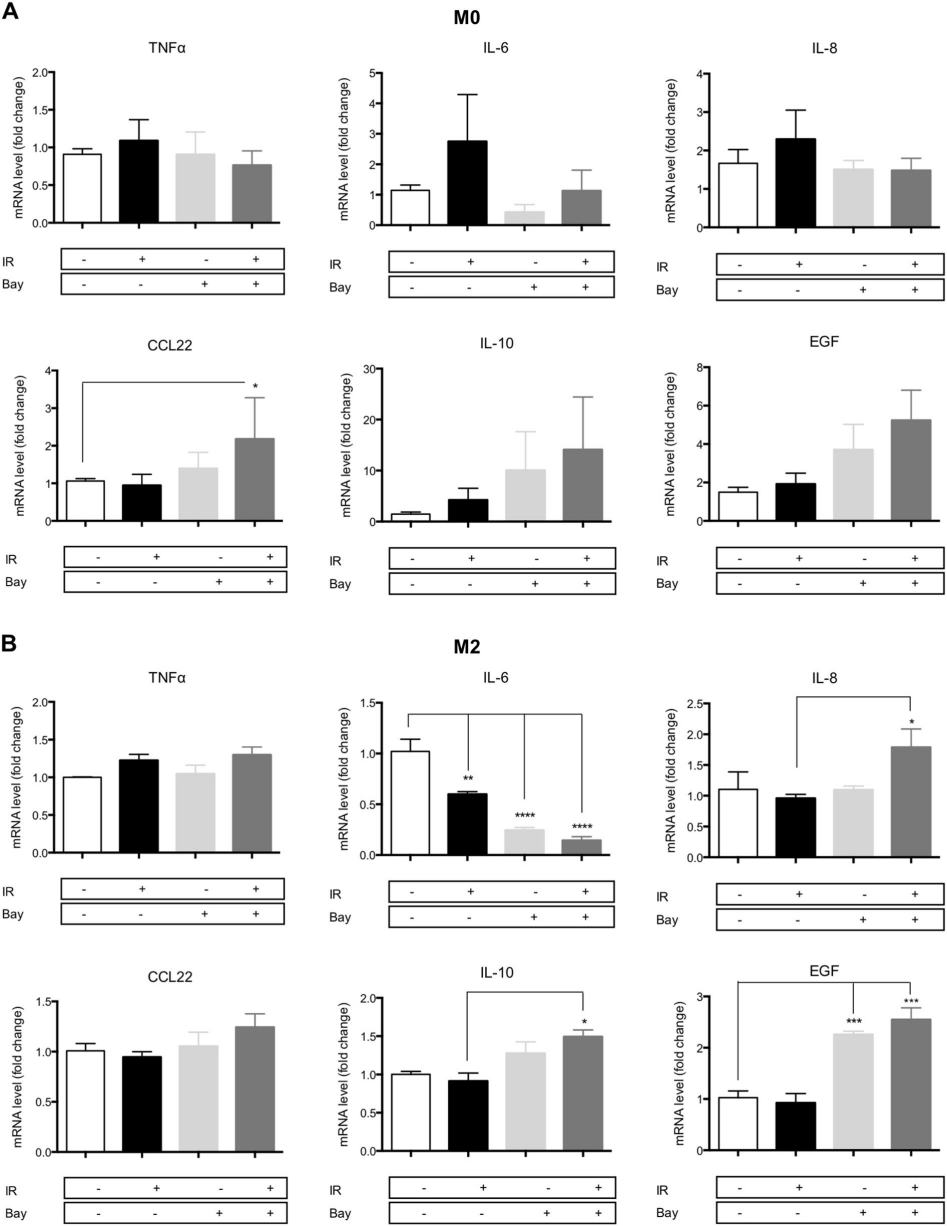
NFκB inhibition reverts proton irradiation-induced macrophage reprogramming. M0 and M2 macrophages were irradiated (IR) with protons (10 Gy) with or without NFκB inhibitor (Bay 11-7082—5 μM). The inhibitor was added 1 h before irradiation for the following 12 h. Macrophage polarization was evaluated by RT-qPCR 12 h after proton irradiation by the analysis of M1 (TNFα, IL-6, IL-8) and M2 (CCL22, IL-10, EGF) marker mRNA level in (**a**) M0 macrophages (*N* = 3, mean ± SD) and **b** M2 macrophages (*N* = 4, mean ± SD). Two-way ANOVA analyses followed by Tukey’s multiple comparison tests were performed on data; *p ≤ 0.05; **p < 0.01; ***p < 0.001

To study the role of NFκB in proton beam-mediated macrophage reprogramming, we assessed the mRNA level of M1 and M2 markers in M0 and M2 macrophages 12 h after Bay 11-7082 (IKK inhibitor) treatment combined with proton irradiation (Fig. 6). Based on preliminary results, we chose to treat macrophages during 12 h with 5 µM of Bay 11-7082 inhibitor. Indeed, for longer incubation time and for higher concentrations, we observed a cell death higher than 20% (data not shown). In accordance with the results from Fig. 4a, the proton irradiation (10 Gy) of M0 macrophages displayed a nonsignificant increase in M1 marker mRNA levels (TNFα, IL-6, and IL-8) 12 h after irradiation while the mRNA expression of M2 markers was not influenced by the irradiation (Fig. 6a). The combination of the IKK inhibitor with the proton irradiation completely inhibited the programming of M0 into M1 phenotype and induced a nonsignificant increase in M2 marker expression (CCL22, IL-10, and EGF). Although Bay 11-8072 treatment alone drove the expression of IL-10 and EGF, the combination of the inhibitor with proton irradiation induced a much higher expression of these genes. The same experiment has been performed for M2 macrophages (Fig. 6b). As it was aforementioned, the irradiation of M2 macrophages had no effect on the M1 marker mRNA levels after 24 h (Fig. 4a) and we detected no change in the TNFα and IL-8 mRNA expression after 12 h (Fig. 6b). However, a decrease in IL-6 expression was noticed in the same condition. When proton irradiation was combined to NFκB inhibition, diverse effects were observed on the expression of M1 markers. No effect was observed on TNFα expression, while the expression of IL-8 surprisingly increased when proton irradiation was combined to Bay 11-7082 treatment. As the activation of NFκB regulates the expression of IL-8, it is surprising to observe an elevation of the IL-8 mRNA level in M2 macrophages irradiated in the presence of Bay 11-7082. However, the transcription of IL-8 is also regulated by other transcription factors, such as the activator protein 1 (AP-1), that could be also activated when NFκB is inhibited in proton-irradiated M2 macrophages^18^. On the other hand, IL-6 expression was strongly decreased by the IKK inhibitor: its expression decreased by four times with Bay 11-7082 alone and by five times with the combination. For M2 markers, proton irradiation alone did not alter the expression of these markers after 24 h (Fig. 4a). In the same line, 10 Gy of irradiation did not change the expression of M2 markers after 12 h (Fig. 6b). However, the combination of NFκB inhibitor to proton irradiation induced a higher expression of M2 markers. The elevation of M2 marker expression was stronger in M2 macrophages exposed to the combined treatment than for macrophages treated with the inhibitor alone. In conclusion, the NFκB inhibitor combined to proton irradiation completely prevented the programming of M0 macrophages towards an M1 phenotype. On the contrary, it promoted the programming of M0 macrophages towards an M2 phenotype. In addition, M2 macrophages reinforce their M2 phenotype when exposed to proton irradiation in combination with Bay 11-7082.

## Discussion

As M2-like TAMs play an important role in tumor promotion and treatment failure, the reprogramming of these cells has been shown to be an efficient way to promote tumor regression. It is already known that macrophages are more radioresistant compared to other cell types^19^. Indeed, the fusion of macrophages with breast cancer cell line (MCF7) resulted in hybrids developing the abilities to resist high radiation doses and to repair DNA damage faster than the parental MCF7 cells^20^. In the present study, we demonstrated the radioresistance of M1 macrophages compared to M0 and M2 phenotypes. Several evidences highlighted the radioresistance of M1 macrophages in the literature. For example, BALB/c mice naturally exhibiting a T_H_1/M1 (T_H_1 lymphocytes and M1 macrophages) response were more radioresistant than C57BL/6 mice naturally exhibiting a T_H_2/M2 response^16^. TNFα play key roles in macrophage radioresistance since an intact TNFα signaling is needed for radioresistance. Indeed, the deletion of tumor necrosis factor receptor 1 or 2 (TFNR1 or TNFR2) in mice rendered macrophages radiosensitive^21^. In contrast to our observations, a recent in vitro study indicated that unpolarized (M0) and M1 macrophages were more sensitive to X-ray irradiation than M2 macrophages. The divergent results could be explained by the use of higher LPS concentration (100 ng/ml instead of 10 pg/ml in the present study) for the polarization process before the irradiation^22^.

The resistance of M1 macrophages to proton irradiation correlated with a higher level of phosphorylated H_2_AX, possibly indicating either more DSBs, or a higher activity of DNA damage repair. Another explanation could rely on the ability of macrophages to detoxify irradiation-induced radicals formed upon H_2_O radiolysis. It is well established that the radioresistance of macrophages is conferred by a high production of anti-oxidative molecules, such as manganese superoxide dismutase (MnSOD). Higher MnSOD expression is associated to a resistance against damaging effects from ROS and reactive nitrogen species^23^. Indeed, the scavenging of ROS by *N*-acetyl-l-tryptophan glucopyranoside (NATG) in J774A.1 macrophages provided a protection against radiation-dependent apoptosis^24^. Furthermore, another in vitro study revealed that mouse peritoneal macrophages polarized towards the M1 phenotype with methionine displayed increased SOD activity and decreased ROS production^25^. In our experiments, the analysis of ROS management revealed a more efficient elimination of ROS in M1 macrophages when challenged with H_2_O_2_ compared to M0 and M2 macrophages (Fig. S2). In conclusion, the radioresistance of M1 macrophages to proton irradiation is related to a higher DNA damage detection. Several causes like a larger antioxidant pool, a more active DNA repair machinery and the differential promoter methylation of genes involved in DNA repair should be taken in considerations for future studies.

Macrophage re-education represents a promising approach to reverse the fate of tumor and generates tumor regression. To our knowledge, this is the first observation of in vitro macrophage reprogramming with particle therapy. Previous studies with conventional radiotherapy (X-rays or γ-rays) revealed an efficient programming of unpolarized macrophages (M0) towards the M1 phenotype but not M2 reprogramming towards M1-like phenotype (for a review, see ref. ^14^). For example, the irradiation of monocyte-derived macrophages with fractionated doses (2 Gy 5 × /week) induced the polarization towards M1-like macrophages as evidenced by a higher expression of proinflammatory genes and a downregulation of anti-inflammatory genes^26^. Another study also revealed the programming of unpolarized macrophages towards a proinflammatory profile after moderate dose of γ-irradiation (2 or 4 Gy)^27^. In the last study, the authors also reported an increased expression of IL-6, IL-8, and TNFα in γ-irradiated (4 Gy) human monocyte-derived macrophages. While the use of X-ray irradiation induced an M1 polarization of unpolarized human monocyte-derived macrophages, Raw 264.7 cells, PMA-differentiated THP-1 cells and peritoneal macrophages^14^, our results indicated that X-ray irradiation failed to program PMA-differentiated THP-1 macrophages in our experimental settings. In our experiments, a resting time period of 24 h allowed a decrease in NFκB gene cluster expression, upregulated during PMA-induced differentiation^28^. This may explain the discrepancy with previously reported results. The release of TNFα by M0, M1, and M2 macrophages after proton irradiation confirms the reprogramming of macrophages to an M1-like phenotype. Indeed, TNFα is a potent anti-M2 polarization factor and strongly correlates with an M1-like phenotype^29^. Inversely, a decrease in mRNA expression of EGF was shown for the three phenotypes upon irradiation. EGF is well known to be involved in both angiogenesis and cell migration, two roles fulfilled by M2 macrophages^30^.

The active heterodimer NFκB p50–p65 is predominant for M1 activation, leading to a proinflammatory profile while the induction of the inactive homodimer NFκB p50–p50 inhibits the expression of proinflammatory genes in M2 macrophages^12, 13^. Our results demonstrated the implication of NFκB p65 subunit in macrophage reprogramming after moderate doses of proton irradiation. In addition, the combination of proton irradiation to an IKK complex inhibitor (Bay 11-7082) completely aborted the re-education towards a M1 phenotype in M0 and M2 macrophages. In general, NFκB is activated in irradiated cancer cells when doses are comprised in the range of 7−10 Gy^31–33^. Similarly to our study, the X-ray irradiation of monocyte-derived macrophages also induced the upregulation of total and phosphorylated p65, from 1 to 6 h after irradiation at 2 Gy and 10 Gy. This was correlated to the reduced expression of anti-inflammatory genes^26^. Furthermore, the whole-body irradiation of RT5 insulinoma bearing mice increased NFκB p65 phosphorylation in tumors, partially explaining the TAM reprogramming after γ-irradiation^15^. Proton irradiation is also more likely to induce more severe DNA damage and higher ROS production compared to photons^34^. These damages activate the DNA repair system, including ATM (ataxia-telangiectasia mutated) kinase, notably responsible for the translocation of NFκB p65 into the nucleus^35^. Hence, ROS production and double DNA strand breaks are strongly linked to NFκB p65 activation^14, 36^. In addition, other studies have demonstrated the activation of NFκB p65 in cancer cells after high LET charged particle irradiation. Indeed, heavy ions with a LET of 100–300 keV/μm showed up to nine times higher potential to activate the NFκB pathway compared to X-rays^37^ while carbon ions with a LET under 73 keV/μm induced an NFκB p65 activation twice as high as the one induced by conventional radiotherapy^38^. For future studies, the use of high LET radiation should be considered, as it can potentiate the activation of NFκB in macrophages and induce apoptosis in cancer cells. However, further investigations are needed to clarify if charged particle therapy may be used for all cancer types. Indeed, constitutive activation of NFκB is associated to tumor growth for several human cancer cells such as breast cancer, colon cancer, prostate cancer, and lymphoid cancer. In cancer cells, NFκB plays key roles in cell survival, notably by regulating the expression of genes involved in cell survival and cell cycle^38^. For conventional radiotherapy, the use of NFκB inhibitors increased the radiosensitivity of many cancer cells^39^. However, no study has been performed yet to assess the balance between NFκB-mediated survival and apoptosis in high LET radiation context. Also, the activation of the immune system by particle therapy could overcome the activation of NFκB in cancer cells. These questions reveal a need for further investigations regarding the use of charged particle therapy in cancer.

Our study provides direct evidences that there could be a selection of M1 macrophages in tumors with proton irradiation, since this phenotype is more radioresistant. Furthermore, we showed that 10 Gy of proton irradiation is able to program M0 macrophages towards M1 phenotype, to enhance the M1 phenotype in M1 macrophages and to initiate an M1 phenotype in M2 macrophages. This re-education is due, at least in part, to NFκB p65 activation. Therefore, our data open new perspectives for macrophage clinical targeting with charged particle therapy. In the future, these results need to be confirmed by in vivo experiments, as other cell types are present in the tumor microenvironment and could influence the response to proton irradiation. It has to be noted that macrophage reprogramming after proton irradiation may not be exclusively associated to NFκB activation. Indeed, the interaction between several pathways (NFκB, MAPK, and IRF/STAT pathways) may be involved in this process and needs to be further investigated. Moreover, metabolism reprogramming has been demonstrated to influence macrophage polarization^40, 41^ and should be taken into account in the further studies.

## Materials and methods

### Differentiation of monocytes to macrophages and macrophage polarization

Human monocytic cell line (THP-1–ATCC TIB-202) were grown in Roswell Park Memorial Institute (RPMI 1640, Gibco #21875034) culture medium containing 10% of heat inactivated fetal bovine serum (Gibco) and supplemented with 10 mM Hepes (Gibco, #15630-056), 2 mM pyruvate (Gibco, #11360-039), 2.5 g/L d-glucose (Merck) and 50 pM β-mercaptoethanol (Gibco, #31350-010). As the proton accelerator used in this study produces a horizontal beam, cells were plated in suitable irradiation chambers, composed of two stainless steel rings with the central holes covered by 3 μm Mylar foils (Goodfellow). THP-1 monocytes were seeded at the center of irradiation chambers, on the Mylar foil, and differentiated into macrophages with 150 nM phorbol 12-myristate 13-acetate (PMA, Sigma P8139). For this step, a cloning cylinder (6.4 mm diameter size, Sigma C3983-50EA) was placed at the center of the chamber and filled with Cell Tak (VWR #354240) for coating. Differentiated THP-1 cells tend to form clusters when seeded on Mylar foil and Cell tak allowed a homogenous monolayer. The cloning cylinders were rinsed with milli Q water and were filled with 190 μl of medium containing 50,000 THP-1 monocytes, penicillin streptomycin 10,000 U/ml (Fisher, #15140122) and PMA for 24 h incubation. The differentiation medium was replaced with RPMI medium for a further 24 h incubation. Macrophages were polarized in M1 phenotype with 10 pg/ml lipopolysaccharides (LPS, Sigma; #8630) and 20 ng/ml interferon γ (IFN-γ, R&D Systems, #285-IF) during 24 h incubation or were polarized in M2 phenotype with 20 ng/ml interleukin 4 (IL-4, R&D Systems, #204-IL) and 20 ng/ml interleukin 13 (IL-13, R&D Systems, #213-ILB) during 48 h incubation as described in ref. ^42^. The gene expression of M1 (IL-1β, TNFα, CXCL10, and IL-6) and M2 (CCL18, CCL22, CD206, and IL-10) markers were analyzed to verify the macrophage phenotype of M0, M1, and M2 macrophages in the irradiation chambers (Fig. 4S).

### Proton irradiation

Four days before irradiation, THP-1 monocytes were differentiated in the irradiation chambers to generate M2 macrophages. To obtain M1 or M0 macrophages, cells were seeded 3 or 2 days respectively before irradiation. Just before proton irradiation, culture medium was changed by CO_2_ independent medium (Gibco #18045054) supplemented with 2 mM l-glutamine and 3.75 g/L of d-glucose. The irradiation chambers were placed at the end of a 2 MV Tandem accelerator (High Voltage Engineering Europa) available at the University of Namur. The experimental set-up and irradiation procedure are described elsewhere^43, 44^. Briefly, an H^+^ homogenous 1 cm^2^ broad beam went through a 1-μm-thick Si_3_N_4_ exit foil. The chambers were placed vertically at 3 mm from the exit window of the accelerator. The linear energy transfer (LET) of protons was set to 25 keV/μm and the dose rate fixed to 2 Gy/min. After irradiation, the chambers were replaced in the CO_2_ incubator until the experiments were performed.

One of the constraints of this set-up is the need to have cells that adhere to the Mylar foil that constitutes the bottom of the irradiation chamber. We cannot use usual plastic flaks/plates since the proton beam generated by our particle accelerator does not have enough energy to go through this material. Most cell lines do not adhere to this material and/or form clusters that do not allow a homogeneous irradiation of all cells. Hence, this work has been performed using THP1-derived macrophages only.

### Determination of cell viability

Because of the Cell Tak, the cells could not be detached from the Mylar foil. Ethidium bromide (1 mg/ml Sigma E8751)−acridin orange (0.3 mg/ml, Sigma A6014) (EBAO) was used at 1:100 dilution in PBS to analyze viability. The cells were stained with EBAO for 5 min, 8 h, or 16 h after irradiation. Cells were then rinsed with PBS and were observed with Olympus stream microscope beyond UV light. Live cells are stained in green while dead cells are stained in orange. Quantifications were performed with Aphelion Lab^®^ software on approximately 1000 cells.

### Immunofluorescence labeling

Cells were fixed for 10 min with paraformaldehyde 4% in PBS, and then permeabilized with triton 0.1% for 5 min. The cells were rinsed three times with PBS–BSA 2% (bovine serum albumin) and incubated overnight at 4 °C with primary antibody in the irradiation chambers. Primary antibodies were 1:800 (γH2AX, BioKe 2577), 1:400 (NFκB p65, Cell Signaling D14E12) or 1:1500 (53BP1, Novus NB100-304) diluted in PBS–BSA 2%. Cells were rinsed three times with PBS–BSA 2% and then incubated for 1 h with secondary antibody 1:1000 diluted (Alexa Fluor 488-conjugated anti-rabbit IgG antibody; Molecular Probes, #A11034) at room temperature. After washing the cells three times with PBS, nuclei were stained with TOPRO-3 (1:80 diluted in RNase solution). Cells were then washed three times with PBS and the Mylar foil was cut around the cell drop and was deposited on a microscope slide, cells on top. A coverslip was mounted using Mowiol (Sigma) above the cells and the cells were observed with a confocal microscope (SP5, Leica). A constant photomultiplier gain value and a constant laser power were used to take the pictures. Quantifications for γH_2_AX intensity or 53BP1 foci were performed using Aphelion Lab^®^ software on approximately 1000 cells. For γH_2_AX, the quantification was performed as followed: mean of (Number of green pixels × intensity of each pixel) / nucleus. Usually, the quantification of γH_2_AX labeling is performed by counting γH_2_AX foci. However, as high doses were used in this study, large numbers of clustered foci were observed and it was impossible to discriminate foci at these doses. Therefore, we choose to quantify the mean intensity of γH_2_AX per nucleus. Apoptotic cells were not taken into account for the quantification. Quantification for 53BP1 foci was performed similarly. Quantification for NFκB p65 was performed as followed: number of cells with higher pixel numbers than mean + 2 standard deviations, representative of cells with high translocation of NFκB p65.

### Relative quantification of mRNA levels

Total RNA was extracted from cells in irradiation chambers with RNeasy micro kit and DNAse protocol (QIAGEN # 74004). Reverse transcription was performed on 1 μg using Transcriptor first strand cDNA synthesis kit (Roche #4309155). The quantitative Real Time PCR was performed using Viia 7 (Thermo Fisher Scientific) with SYBRGreen PCR Master Mix (Applied Biosystems, #4309155) and primers (IDT, 300 nM). 40S ribosomal protein S9 (RPS9) was selected as the housekeeping gene for normalization, based on its constant expression within all samples. The primers used for RT-qPCR are summarized in supplementary information (table S1).

### Cytokine quantification

Secreted cytokine (IL-6, CXCL-8/IL-8, and TNFα) analysis was assessed using ELISA kit, according to the manufacturer’s recommendations (Quantikine, R&D Systems D6050, D8000C and DTA00C). Culture media were diluted in CO_2_ independent medium 5 and 50 times for TNFα and IL-8, respectively, while the culture medium was not diluted for IL-6. For all samples, concentrations were normalized by total protein (μg proteins/ml) determined by Folin method.

### IKK inhibition

Bay 11-7082 inhibitor (3-((4-methylphenyl)sulfonyl)-(2E)-propenenitrile; Selleckchem S2913) selectively and irreversibly inhibits the phosphorylation of IκB-α and then prevents the NFκB activation. This inhibitor was added at 5 μM in CO_2_ independent medium 1 h before irradiation and the cells were incubated for 12 h after irradiation in the presence of the inhibitor. TNFα (20 ng/ml, R&D Systems #210-TA-020) was used as a positive control to verify the nuclear translocation of NFκB p65. In order to validate the inhibition of p65 nuclear translocation in our model, cells were incubated with TNFα and Bay 11-7082 (Fig. 5S).

### Statistical analysis

Statistical analyses were performed using Graph Pad Prism software. Data are reported as mean ± 1SD of *N* independent experiments. A *p* value of <0.05 was considered as significant.

## Electronic supplementary material

GGenard-Supplementary data
