# Supplementary material for: Proton irradiation orchestrates macrophage reprogramming through NFκB signaling
Source: Cell Death Dis. 2018 Jun 27;9(7):728. doi: 10.1038/s41419-018-0757-9 (PMC6021396; doi:10.1038/s41419-018-0757-9)
Supplement: Supplementary file 1 — GGenard-Supplementary data [file 41419_2018_757_MOESM1_ESM.pdf]

# Supplementary Data

**MNAse I sensitivity assay**

Heterochromatin content was evaluated in M0, M1 and M2 polarized THP-1 cells (3.6 x $10^6$) as described in (1). Briefly, cells were homogenized in RBS buffer (10 mmol/l Tris-HCl (pH 7.4), 10 mM NaCl, 3 mM$MgCl_2$, 0.5% NP-40) for 15 min on ice. Samples were then centrifuged at 4°C for 5 min at 1400 *g*. The medium was discarded and the pellet was washed twice with RBS buffer. Samples were then digested with 2.5 U of micrococcal nuclease in digestion buffer (15 mM Tris-HCl (pH 7.5), 60 mM KCl, 15 mM NaCl, 1 mM $CaCl_2$, 3 mM $MgCl_2$, 20% glycerol, 15 mM β-mercaptoethanol) for 30 seconds, 1, 2 or 5 minutes. Stop solution (50 mM Tris (pH 7.5), 150 mM NaCl, 50 mM EDTA, 0.3% sodium dodecyl sulfate (SDS)) was used to stop digestion in samples. DNA was extracted using phenol/chloroform method and was precipitated with ethanol 100%. DNA was washed once with 70% ethanol, resuspended in $H_2O$ and was separated using 1.2% agarose gel.

**Reactive oxygen species measurement**

ROS management in M0, M1 and M2 THP-1 cells was evaluated using a fluorescent probe, CM-$H_2$DCFDA (DCF-DA). THP-1 cells were differentiated and polarized in 6 well plates (8 x $10^5$ per well). After polarization, cells were rinsed with PBS. Cells were trypsinized and centrifuged at 1000 rpm for 7 minutes. Cells were resuspended in 2% heat inactivated serum – PBS and were centrifuged at 4°C for 5 minutes at 1200 rpm. Macrophages were incubated with DCFDA dye (10 μM) for 20 minutes at 37°C and centrifuged again at room temperature for 5 minutes at 1200 rpm. Cells were exposed to different concentrations (0.25, 1 and 5 μM) of hydrogen peroxide solution ($H_2O_2$), for 15 minutes at 37 °C. After rinsing the cells in 2% heat inactivated serum – PBS, cells were resuspended in cold PBS. Fluorescence was measured by flow cytometry using FACS calibur (BD Biosciences).

**X-ray irradiation**

Four days before irradiation, 800 000 THP-1 monocytes were differentiated in 6-well plates to generate M2 macrophages. To obtain M1 or M0 macrophages, cells were seeded three or two days respectively before irradiation. Just before X-ray irradiation, culture medium was changed by $CO_2$ independent medium (Gibco #18045054) supplemented with 2 mM L-glutamine and 3.75 g/L of D-glucose. Photon beam (2 Gy/min) was produced with X-RAD 225 kV (Precision X-ray) irradiator and the cells were seeded in six-well plates, positioned at 30 cm from the source. After irradiation, the 6-well plates were replaced in the $CO_2$ incubator until the samples were recovered.

| Gene | Forward | Reverse |
|---|---|---|
| **CCL22** | CACTTCTACTGGACCTCAGAC | AGTAGGCTCTTCATTGGCTCA |
| **EGF** | ATGTCCTGCCCTCAACC | GGTTGCATTGACCCATCTGC |
| **IL-6** | CCTGAACCTTCCAAAGATGGC | CACCAGGCAAGTCTCCTCATT |
| **IL-8** | TCTGTGTGAAGGTGCAGTTTT | GGGGTGGAAAGGTTTGGAGTA |
| **IL-10** | TTCCCTGTGAAAACAAGAGCAA | GTAGATGCCTTTCTCTTGGAGCTTA |
| **RPS9** | CTGGATGAGGGCAAGATGAAG | GTCTGCAGGCGTCTCTCTAAGAA |
| **TNFα** | CTGCACTTTGGAGTGATCGG | TCAGCTTGAGGGTTTGCTAC |

**Table S1**. Primers used for RT-qPCR

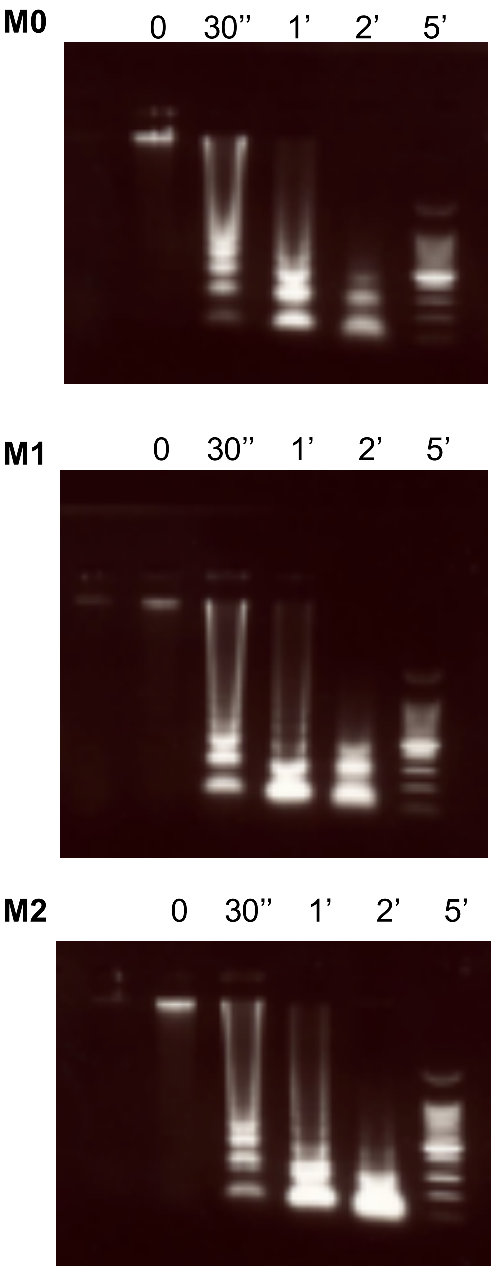


**Figure S1. Heterochromatin content in M0, M1 and M2 macrophages**
Heterochromatin content was evaluated by MNAse assay. DNA was extracted from M0, M1 and M2 macrophages and then exposed to MNAse enzyme for 30 seconds, 1, 2 or 5 minutes. Resulting fragments were separated by electrophoresis. Representative images show the migration profile of fragmented DNA in M0, M1 and M2 macrophages after MNAse treatment (N=5).

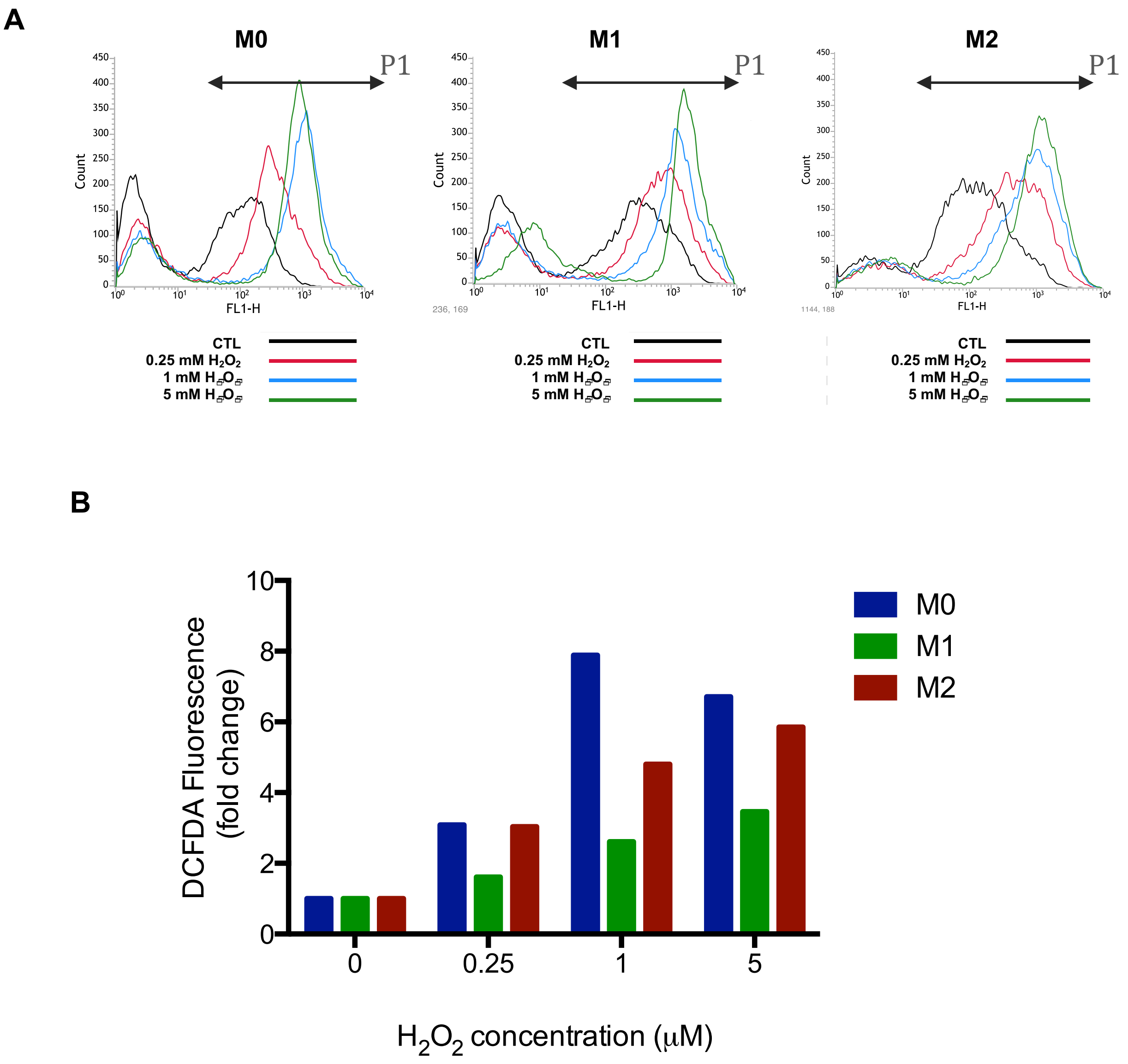


**Figure S2. Differential ROS management in M0, M1 and M2 macrophages**
M0, M1 and M2 macrophages were treated with different $H_2O_2$ concentrations (0, 0.25, 1 and 5 μM) and ROS content was detected by flow cytometry (DCFDA fluorescence). **A**, Evaluation of ROS content in M0, M1 and M2 macrophages after exposure to different $H_2O_2$ concentrations. **B**, Quantification of the mean fluorescence (ROS level) in macrophages in gated population (P1). Results are normalized to the untreated condition (N=1).

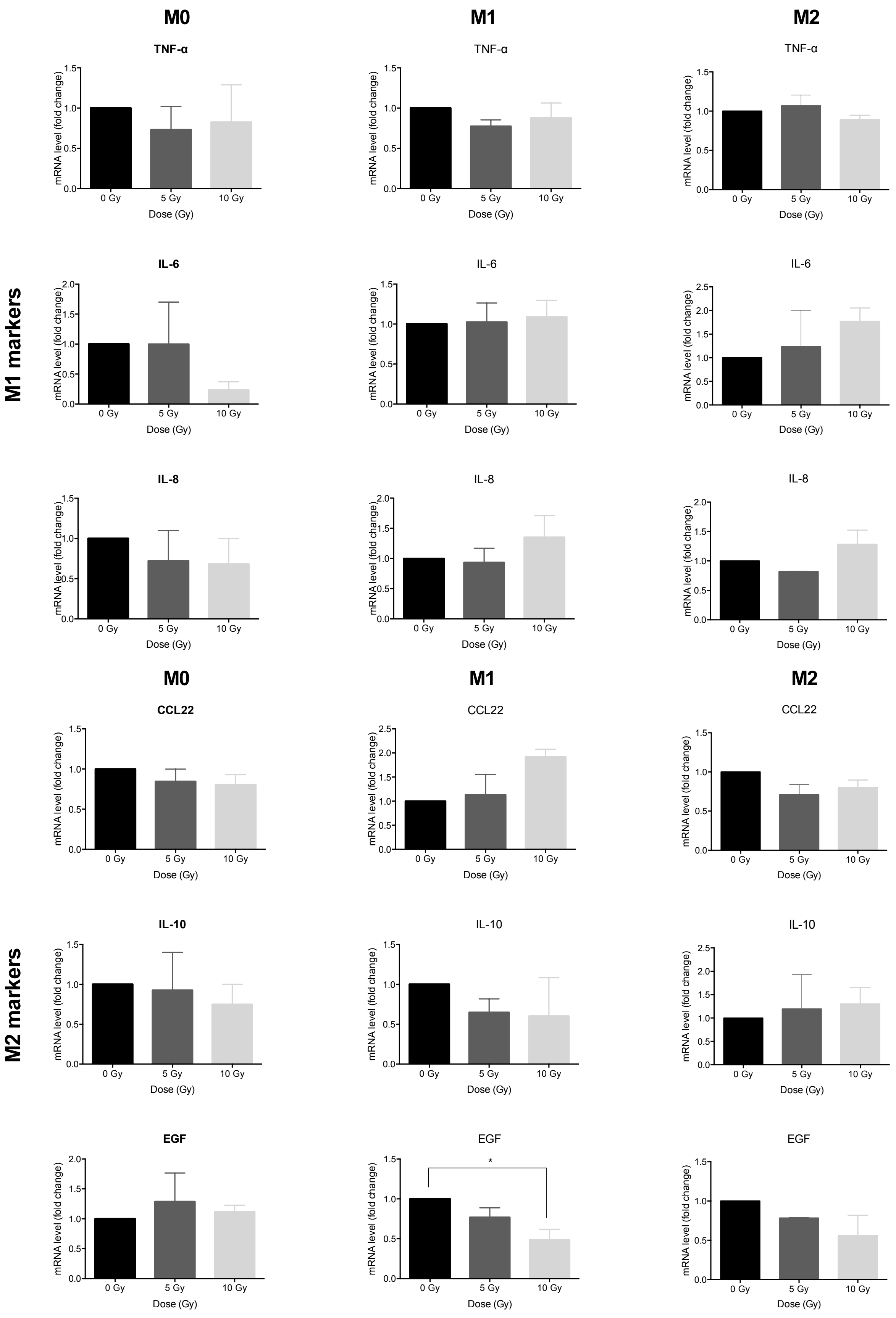
M0
M1
M2
M1 markers
TNF-α
IL-6
IL-8
M2 markers
CCL22
IL-10
EGF
mRNA level (fold change)
0 Gy
5 Gy
10 Gy
Dose (Gy)
*

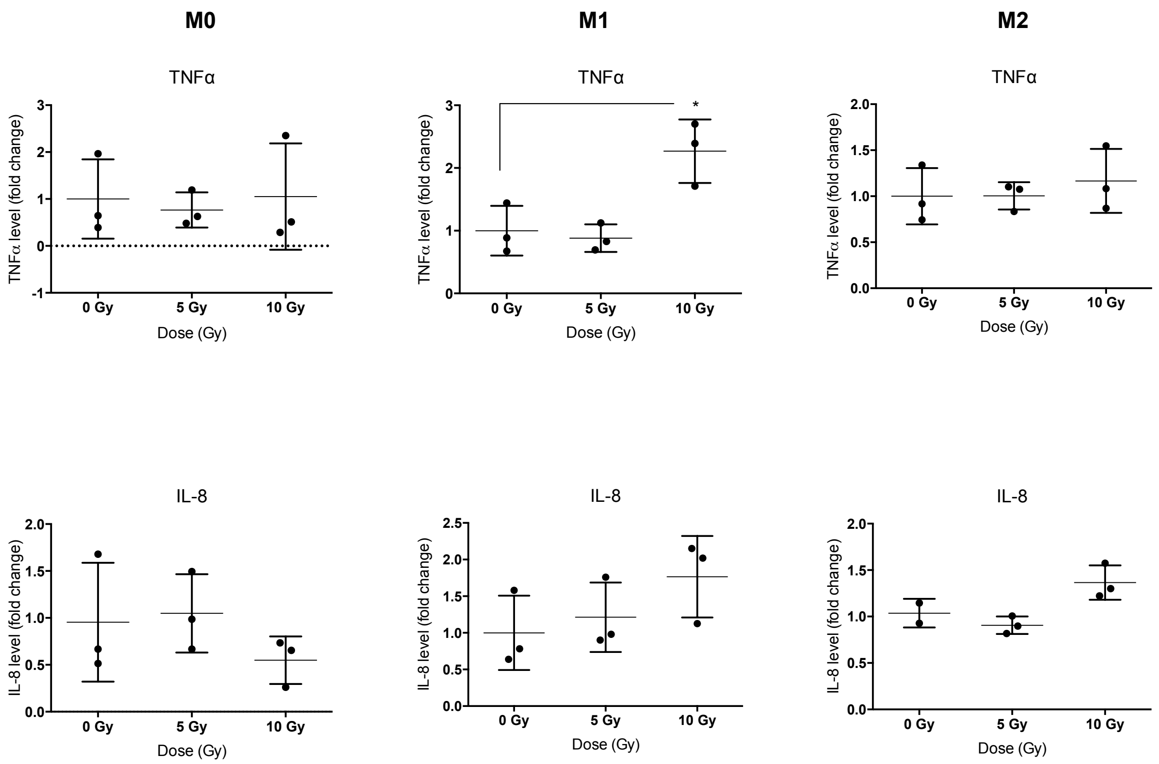


**Figure S3**. **X-ray irradiation does not induce macrophage reprogramming**
M0, M1 and M2 macrophages were irradiated with different doses of X-rays (0, 5 and 10 Gy) **A,** 24h after X-ray irradiation, mRNA levels of M1 (TNFα, IL-6, IL-8) and M2 (CCL22, IL-10, EGF) markers were assessed by RT-qPCR (N=3, mean ± SD). One-way ANOVA analyses followed by Dunnett's multiple comparison tests were performed to evaluate the significance (* $p \leq 0.05$; ** $p < 0.01$; *** $p < 0.001$). **B,** 24h after X-ray irradiation, TNFα and IL-8 secretion was evaluated by ELISA. Results are expressed in pg/ng of proteins and are normalized to the non-irradiated condition (fold change) for each macrophage phenotype (N=3, mean ± SD). One-way ANOVA analyses followed by Kruskal Wallis multiple comparison tests were performed on data (* $p \leq 0.05$; ** $p < 0.01$; *** $p < 0.001$).

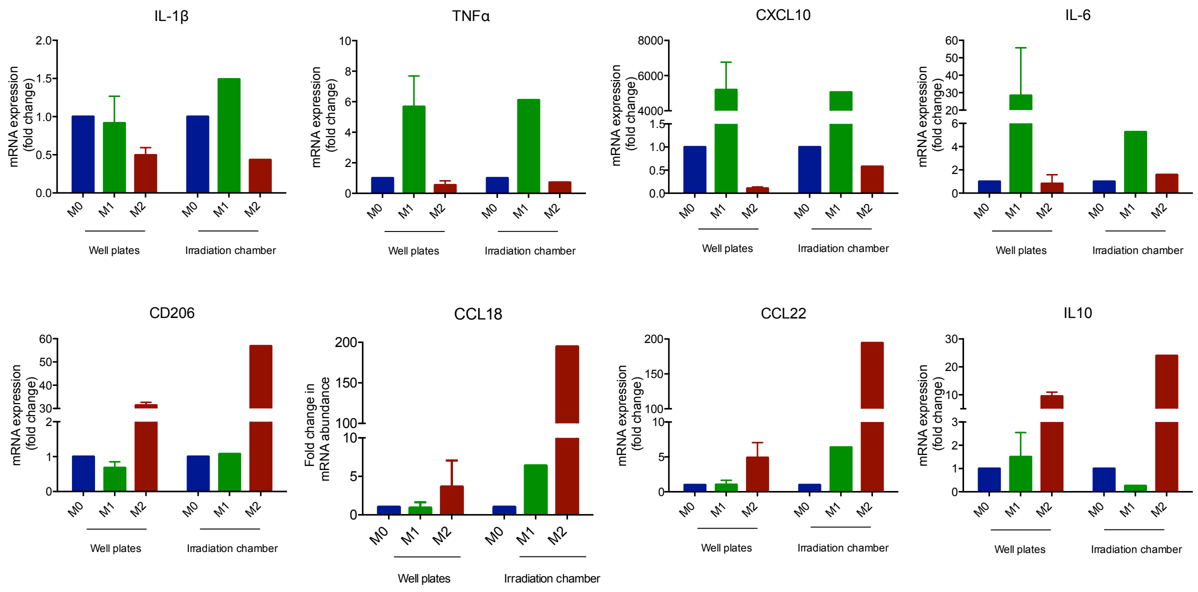


**Figure S4. Evaluation of macrophage phenotype after differentiation and polarization in the irradiation chambers**

THP-1 cells were differentiated and polarized into M0, M1 and M2 macrophages in conventional well plates and irradiation chambers. mRNA levels of M1 (IL-1β, TNFα, CXCL10 and IL-6) and M2 (CD206, CL18, CCL22 and IL-10) markers were assessed by RT-qPCR in M0 (blue), M1 (green) and M2 (red) macrophages (N=2 for well plate; N=1 for irradiation chamber).

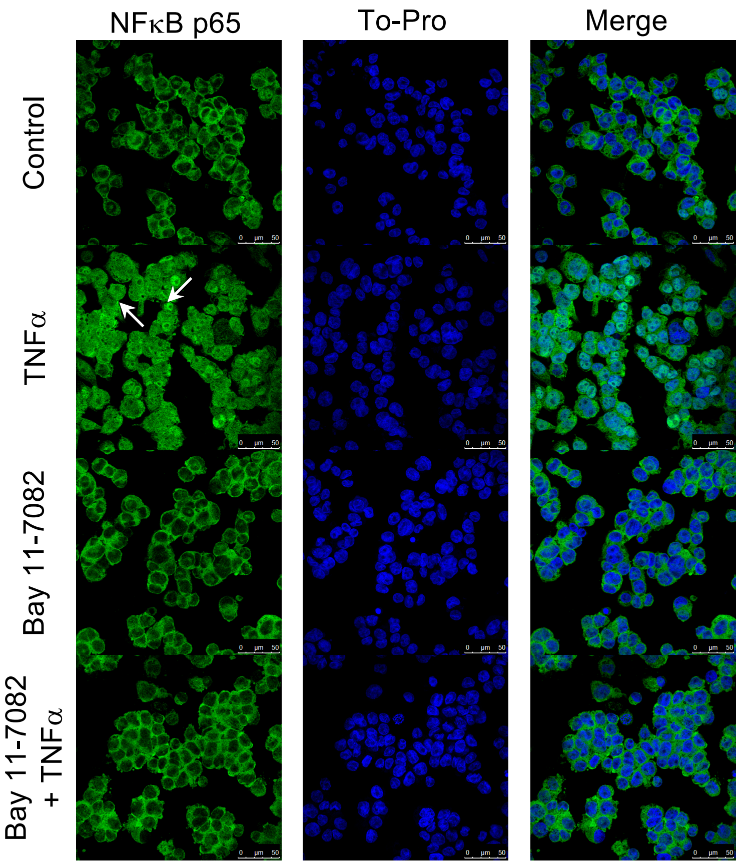


**Figure S5. Inhibition of nuclear NFκB p65 translocation after Bay 11-7082 exposure**
M0 macrophages were exposed to TNFα (20 ng/ml) or to Bay 11-7082 inhibitor (5 μM) as well as to the combination of both TNFα and Bay 11-7082 for 12h. The nuclear translocation of NFκB p65 was evaluated by immunofluorescence staining. Representative immunofluorescence staining images display NFκB p65 in green and nuclei in blue (N=1).

1. Peixoto P, Castronovo V, Matheus N, Polese C, Peulen O, Gonzalez A, et al. HDAC5 is required for maintenance of pericentric heterochromatin, and controls cell-cycle progression and survival of human cancer cells. Cell Death Differ. 2012;19(7):1239-52.
